# Is Initiating NOACs for Atrial Arrhythmias Safe in Adults with Congenital Heart Disease?

**DOI:** 10.1007/s10557-017-6745-y

**Published:** 2017-08-07

**Authors:** Hayang Yang, Berto J. Bouma, Barbara J. M. Mulder, On behalf of The Non vitamin K antagonist Oral anticoagulants for ThromboEmbolic prevention in adult congenital heart disease (NOTE) investigators, J.F. Heidendael, G. Veen, T.C. Konings, G.T.J. Sieswerda, F.J. Meijboom, M.C. Post, A. van Dijk, W. Budts, M. Morissens, M. Ladouceur, D. Tobler, M. Schwerzmann, T. Rutz, J. Bouchardy, M. Greutmann, G. Scognamiglio, K. Skoglund, C. Christersson, L. Gumbiene, M. Laukyte, P. Khairy, J. Aboulhosn, G. Veldtman, G. Webb, C.S. Broberg, A.R. Opotowsky, K. Shafer, S.F. Tsai, T. Moe, K. Niwa, A. Mizuno

**Affiliations:** 1 Department of Cardiology, Academic Medical Center, University of Amsterdam, Room B2-240, Meibergdreef 9, 1105 AZ Amsterdam, The Netherlands; 2 Interuniversity Cardiology Institute of the Netherlands-Netherlands Heart Institute, Utrecht, The Netherlands

**Keywords:** Congenital heart disease, Atrial arrhythmia, Anticoagulation, Non vitamin K antagonist oral anticoagulant, Thromboembolic event, Bleeding

## Abstract

**Background:**

In recent years, non-vitamin K antagonist (VKA) oral anticoagulants (NOACs) have been increasingly prescribed to adults with congenital heart disease (CHD) and atrial arrhythmias without good evidence for either safety or efficacy. To address this gap, we initiated an ongoing prospective global registry (NOTE: **n**on-vitamin K antagonist **o**ral anticoagulants for **t**hrombo**e**mbolic prevention in patients with congenital heart disease). Using the NOTE registry data, the present study aimed to evaluate the occurrence of any adverse events during the initiation phase (first 30 days) of NOACs in adults with CHD and atrial arrhythmias.

**Methods and Results:**

For this prospective observational study, 99 adults with CHD and atrial arrhythmias (median age 49 years [IQR 38-61], 53% male) who initiated NOACs at or after the inclusion point were analysed. Thromboembolic events, major bleeding and other minor adverse events were assessed after the first 30 days since the initiation of NOACs. In 54 patients transitioning from VKA to NOACs, 8 minor adverse events (5 minor bleeding; 3 side-effects; 1 drop-out due to minor bleeding) occurred within 30 days after the transition. No adverse events were reported in 46 VKA-naive patients within 30 days after the initiation of NOACs.

**Conclusions:**

Initiation of NOACs and transition from VKA to NOACs seem to be safe during the first month, without major adverse events and with only limited minor side effects in adults with CHD and atrial arrhythmias. This global ongoing prospective registry enables precise collection of important clinical information in real-world adults with CHD, managed with NOACs.

**Electronic supplementary material:**

The online version of this article (doi:10.1007/s10557-017-6745-y) contains supplementary material, which is available to authorized users.

## Introduction

Atrial arrhythmias are prevalent in 15% of adults with congenital heart defects (CHD) and are associated with increased risk for thromboembolism [1]. The most recently introduced oral anticoagulants, non-vitamin K antagonist oral anticoagulants (NOACs) have been shown to be associated with an equal or reduced risk for thromboembolism and major bleeding compared with vitamin K antagonist (VKA) in the general population with non-valvular atrial arrhythmias [2]. However, it is uncertain whether results from that population are applicable in CHD patients, since hearts of CHD patients are structurally different. Nevertheless, NOACs are attractive alternatives for VKA in this group of predominantly young patients, as they may be compromised in their daily life by ongoing international normalized ratio (INR) monitoring and dose adjustments. To date, few data have been published on the rates of thromboembolism and major bleeding under the use of NOACs in adults with CHD with atrial arrhythmias [3]. The utility of these data are hampered by a limited sample size and a short follow-up period.

Accordingly, safety of NOACs needs to be assessed in adults with CHD. For this purpose, we initiated a worldwide prospective registry of efficacy and safety of non-vitamin K antagonist **o**ral anticoagulants for **t**hrombo**e**mbolic prevention in patients with congenital heart disease; NOTE registry (https://note.reports.nl; ClinicalTrials.gov registration: NCT02928133). We present our first data on the safety of initiation of NOACs and transition from vitamin K antagonists to NOACs during the first 30 days in adult CHD patients with atrial arrhythmias.

## Methods

In the NOTE registry, adults with CHD using NOACs for thromboembolic prevention have been enrolled from April 2014 onward. The NOTE study protocol conforms to the ethical guidelines of the 1975 Declaration of Helsinki and was approved by research committees of all participating medical centres. Informed consent was obtained from all individual participants included in the study.

This is a prospective cohort study, using data obtained from the NOTE registry. We analysed all patients using NOACs for atrial arrhythmias. We excluded the patients who initiated NOACs prior to the time of enrolment or used NOACs for other indications than atrial arrhythmias. The patients were included as they presented at the participating institutions or were identified by using a national registry. The choice of NOAC type was at the discretion of the patient’s cardiologist without a specific protocol. At inclusion, demographics and pre-defined clinical data were collected. The time period of evaluation of the safety of NOACs initiation was set as within 30 days after the initiation of NOACs. Follow-up took place at 180 ± 28 days, 1 year ±28 days, and 2 years ±28 days, coinciding with their routine outpatient clinical visit or telephone contact. At such follow-up point, evaluation of pre-defined end points was conducted by an investigator or a site coordinator, who was trained on the protocol of the NOTE registry. The major adverse events were thromboembolism (ischemic cerebrovascular accident iCVA, transient ischemic attack TIA, systemic or pulmonary embolism or intracardiac thrombosis) and major bleeding (significant bleeding necessitating hospitalization/interventions/≥2 units of packed cells, and/or with a haemoglobin drop >1.24 mmol/L and/or bleeding that was fatal or occurred in the following critical sites: intra-cranial, intra-spinal, intra-ocular, pericardial, intra-articular, intra-muscular with compartment syndrome) according to the International Society on Thrombosis and Haemostasis criteria [4]. As secondary endpoints, we registered other adverse events (minor bleeding event, mortality, hospitalization, interventions).

The VKA-experienced group consisted of the patients who used VKA at the time of transitioning to NOAC. The rest of the group was defined as the VKA-naive group. Any differences between the VKA-experienced group and VKA-naive group, and between the group with minor bleeding and the group without minor bleeding were analysed using unpaired t-tests and reported as median with IQR or frequencies in % as appropriate. Analyses were performed with SPSS version 21.0 (IBM Corp., Armonk, NY, USA). A *p*-value below 0.05 was considered statistically significant.

## Results

Through collaboration with the International Society for Adult Congenital Heart Disease (ISACHD), there are currently 31 participating medical institutions worldwide and enrolment has started in 26 of these institutions for this ongoing multicenter prospective registry. So far, 245 adults using NOACs were recruited in the NOTE registry.

**Fig. 1:**
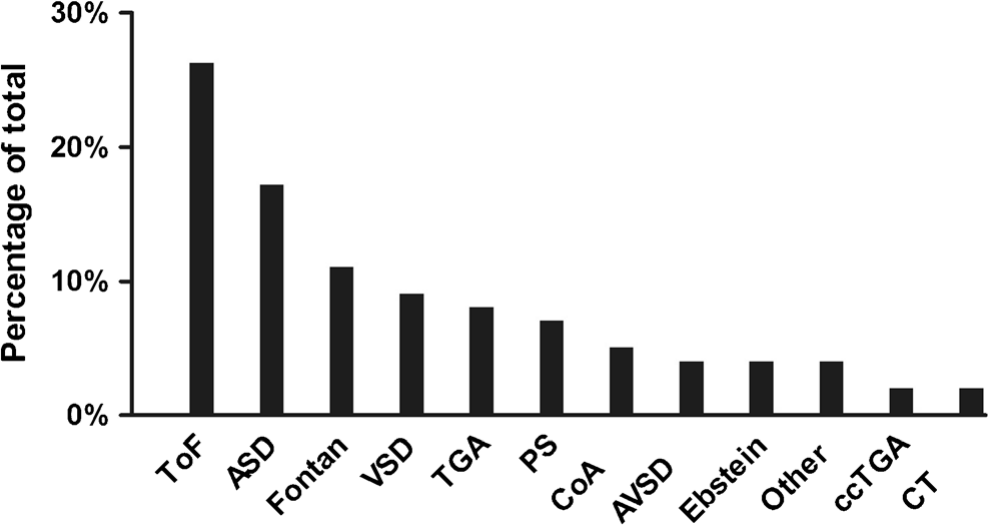
Distribution of congenital heart defects. Abbreviations: ToF-tetralogy of Fallot; ASD-atrial septal defect; Fontan-Fontan circulation; VSD-ventricular septal defect; TGA-transposition of the great arteries; PS-pulmonary valve stenosis; CoA- aortic coarctation; AVSD-atrioventricular septal defect; Ebstein-Ebstein’s anomaly; Other- double-outlet right ventricle, pulmonary atresia, bicuspid aortic valve, Marfan syndrome; ccTGA-congenitally corrected transposition of the great arteries; CT-cor triatriatum

For the present study, 146 patients were excluded from the analysis according to the exclusion criteria. A total of 99 adults (Table 1; median age 49 years [IQR 38–61], 53% male) represent the study cohort for this analysis. The cohort consists of a wide variety of CHD types (Fig. 1; complex 29%, moderate 56%, simple 15%). At the time of transition to NOACs (=baseline), 41% of the patients were not on any anti-thrombotic therapy, 4% were on aspirin and 55% used VKA (2 patients concomitant aspirin; median treatment period 7.7 years [IQR 2.5–10.6]). All patients started NOACs (apixaban 62%; rivaroxaban 29%; dabigatran 9%) for atrial arrhythmias. The most common types of atrial arrhythmias were atrial fibrillation (60%) and atrial flutter or intra atrial re-entry tachycardia (44%). Regarding the CHA_2_DS_2_-VASc and HASBLED score, 57% and 88% of the patients had a score of 0 or 1, respectively. Overall, 11% had Fontan circulation (atriopulmonary connection *n* = 6; lateral tunnel or intracardiac conduit *n* = 4; extracardiac conduit *n* = 1), 7% pulmonary hypertension and 33% had a history of heart failure defined by clinical signs or low systolic function by imaging (Table 1). Twelve percent of the patients previously experienced an iCVA or TIA, 3% deep venous thrombosis, 3% intracardiac thrombosis, 2% pulmonary embolism, 1% myocardial infarction, and 9% had prior major bleeding. Compared with the VKA-naive group, the VKA-experienced group had higher HASBLED score (median 0 [IQR 0–1] vs. 1 [IQR 0–1], *p* = 0.002) and higher prevalence of prior major bleeding (2.2% vs. 14.8%, *p* = 0.030).

**Table 1:** Baseline characteristics

	**All (** ***n*** **= 99)**	**VKA (** ***n*** **= 54)**	**VKA-naive (** ***n*** **= 45)**	***p*** **-value**
Age at inclusion, y	48.8 (38–61)	47.3 (38–61)	52.0 (37–61)	0.784
Male, n(%)	52 (53)	24 (44)	28 (62)	0.078
Severity of congenital heart defect, n (%)				
Simple	15 (15)	8 (15)	7 (16)	0.918
Moderate	55 (56)	28 (52)	27 (60)	0.417
Complex	29 (29)	18 (33)	11 (24)	0.333
Fontan circulation	11 (11)	9 (17)	2 (4)	0.054
Pulmonary hypertension	7 (7)	3 (6)	4 (9)	0.519
Median CHA_2_DS_2_-VASc	1 (0–2)	1 (0–2)	1 (0–2.5)	0.632
Median HASBLED	0 (0–1)	1 (0–1)	0 (0–1)	0.002
Cardiovascular history, n(%)				
Stroke or TIA	12 (12)	7 (13)	5 (11)	0.779
Pulmonary embolism	2 (2)	1 (2)	1 (2)	0.941
Deep venous thrombosis	3 (3)	1 (2)	2 (4)	0.454
Intracardiac thrombosis	3 (3)	3 (6)	0	0.095
Myocardial infarction	1 (1)	1 (1)	0	0.359
Major bleeding	9 (9)	8 (15)	1 (2)	0.030
Heart failure*	33 (33)	20 (37)	13 (29)	0.392
Hypertension	24 (24)	11 (20)	14 (30)	0.319
Diabetes mellitus	8 (8)	4 (7)	4 (9)	0.788

Values are presented as median (IQR) or counts(%). *Heart failure is defined as the presence of signs and symptoms of either right (elevated central venous pressure, hepatomegaly, dependent oedema) or left ventricular failure (exertional dyspnoea, cough, fatigue, orthopnoea, paroxysmal nocturnal dyspnoea, cardiac enlargement, crackles, gallop rhythm, pulmonary venous congestion) or both, confirmed by non-invasive or invasive measurements demonstrating objective evidence of cardiac dysfunction

Abbreviations: VKA, vitamin K antagonist-experienced group; VKA-naive, vitamin K antagonist naive group; CHA2DS2-VASc, stroke risk factor scoring system in which 1 point is given for heart failure, hypertension, age 64–74 years, diabetes mellitus, history of vascular disease, female sex and 2 points are given for age ≥ 75 years, history of stroke/TIA/thromboembolism; HASBLED, bleeding risk factor scoring system in which 1 point is given for uncontrolled hypertension, abnormal renal or liver function, history of stroke or bleeding, labile international normalized ratio, age > 65 years, use of nonsteroidal anti-inflammatory drug or antiplatelet agents or alcohol; NOAC, new oral anticoagulant; TIA, transient ischemic attack

Regarding the safety during the first 30-day follow-up after initiation of NOACs, none of the patients experienced a thromboembolic event or a major bleeding complication, either in the VKA-naive group or in the VKA-experienced group. In the VKA-experienced group (*n* = 54), 9% of the patients reported minor bleeding, most often epistaxis (*n* = 3), hematoma (*n* = 1) and gingival bleeding (*n* = 1), within the first 30 days after the transition. Due to frequent epistaxis, a 42-year old female with coarctation of the aorta, ceased NOAC therapy within 30 days and switched back to VKA. Other side effects such as dizziness, headaches, nausea and weakness were also reported (*n* = 3) in this group. All patients who reported minor adverse events (*n* = 8) were in the VKA-experienced group and were relatively young (< 65 yr) and had moderate or severe CHD; none had a Fontan circulation. No adverse events were reported by patients in the VKA-naive group (*n* = 45). The prevalence of higher HASBLED score (≥2) was similar between the group with minor bleeding and the group without minor bleeding (12.8% vs. 0%, *p* = 0.394).

## Discussion

Initiation of NOACs and transition from VKA to NOACs appeared to be safe for at least the short-term, without major adverse events and with limited minor side effects in 99 adults with CHD and atrial arrhythmias.

Current guidelines state that NOACs are only considered in patients with a simple form of CHD due to lack of data in patients with moderate or complex forms of CHD [5]. Our findings show reassuring short term safety results in a cohort largely consisting of moderate or complex forms of CHD (85%), including patients with Fontan circulation (11%) who are prone to thromboembolism and bleeding. This result may be due to the low median CHA_2_DS_2_-VASc score, HASBLED score and young age in this cohort. Furthermore, recent studies show mixed results on the validity of CHA_2_DS_2_-VASc and HASBLED scores in adults with CHD and atrial arrhythmias [6, 7]. However, due to the presence of their structural heart defects, adults with CHD are considered to have other risk factors for stroke than just the CHA_2_DS_2_-VASc score.

Notably, all patients with minor adverse events were VKA-experienced and had moderate to severe type of CHD. This may be related to the higher HASBLED score and higher prevalence of prior major bleeding in the VKA-experienced group compared with the VKA-naive group. However, a higher HASBLED score (≥2) at baseline was not related to higher minor bleeding risk during the follow up.

This study is limited by the modest sample size, number of events over a short-term follow-up and the observational design. Therefore, the data should be interpreted with caution pending validation in other cohorts with larger sample sizes. Furthermore, due to the low number of events in this study, we were unable to perform regression analysis to determine whether CHD complexity is a predictor of adverse events. We expect to address many of these limitations in the future with this ongoing global registry and welcome new participants to join us ( https://note.reports.nl ).

## Conclusions

Initiation of NOACs and transition from VKA to NOACs seem to be safe during the first month, without major adverse events and with limited minor side effects in adults with CHD and atrial arrhythmias. This global ongoing prospective registry enables precise collection of important clinical information in real-world adults with CHD, managed with NOACs.

## Electronic supplementary material

ESM 1 (DOC 33 kb)
