# Supplementary material for: Is Initiating NOACs for Atrial Arrhythmias Safe in Adults with Congenital Heart Disease?
Source: Cardiovasc Drugs Ther. 2017 Aug 7;31(4):413–7. doi: 10.1007/s10557-017-6745-y (PMC5591797; doi:10.1007/s10557-017-6745-y)
Supplement: Supplementary file 1 — (DOC 33 kb) [file 10557_2017_6745_MOESM1_ESM.doc]

**Appendix I. Definitions of outcome**

*Thromboembolism* was defined as ischemic cerebrovascular accident (iCVA), transient ischemic attack (TIA), systemic or pulmonary embolism or intracardiac thrombosis.

- iCVA was defined as a non-traumatic focal neurologic deficit lasting ≥24 hours with an absence of intracerebral hemorrhage or tumor on the first computed tomographic scan or magnetic resonance imaging obtained after the onset of the deficit.
- TIA was defined as a non-traumatic abrupt onset of a focal neurologic deficit lasting <24 hours.
- Systemic embolism was defined as a clinical history consistent with an acute loss of blood flow to a peripheral artery (or arteries) supported by evidence of embolism from surgical specimens, autopsy, angiography, vascular imaging, or other objective testing.
- Intracardiac thrombus was defined as a discrete echo dense mass in the ventricle with defined margins that are distinct from the endocardium and seen throughout systole and diastole.
- Pulmonary embolism was defined as an acute, symptomatic, objectively verified thrombosis of pulmonary arteries (with ventilation-perfusion lung scanning, angiography, or spiral computed tomography of pulmonary arteries).
- Deep venous thrombosis was defined as an acute, symptomatic, objectively verified proximal deep-vein thrombosis of the legs (with use of compression ultrasonography or venography of leg veins).

*Major bleeding* was defined as a significant bleeding necessitating hospitalization/interventions/≥2 units of packed cells, and/or with a Hb drop > 1,24 mmol/L and/or bleeding that was fatal or occurred in the following critical sites: intra-cranial, intra-spinal, intra-ocular, pericardial, intra-articular, intra-muscular with compartment [ISTH bleeding criteria].

*Intracranial bleeding* was defined as a non-traumatic focal neurologic deficit lasting ≥24 hours with a sign of intracerebral hemorrhage in a cerebral imaging study (computed tomographic scan or magnetic resonance imaging).

*Minor bleeding was* defined as all acute clinically overt bleeding events not meeting criteria for major bleeding.
